# Supplementary material for: The role of N-glycosylation in spike antigenicity for the SARS-CoV-2 gamma variant
Source: Glycobiology. 2023 Dec 4;34(2):cwad097. doi: 10.1093/glycob/cwad097 (PMC10969516; doi:10.1093/glycob/cwad097)
Supplement: Supplementary_Material_1_Directory_of_files_within_the_MassIVE_dataset_MSV000091533_cwad097 [file supplementary_material_1_directory_of_files_within_the_massive_dataset_msv000091533_cwad097.pdf]

The *N*-occupancy and glycoproteomic datasets generated and analysed during the current study are available in the MassIVE (Mass Spectrometry *I*nteractive Virtual Environment) repository, [ftp://MSV000091533@massive.ucsd.edu; Username for web access: MSV000091533_reviewer; Password: Gamma_Glyco].

Dataset **MSV000091533**

RAW MS FILES (198 files)

Occupancy PNGAse F and Glycosylation Validation
- Occupancy Glycosylation Validation (27 samples, 27 RAW files, file name: *Number_Replicate_G_Protein*)
- Occupancy PNGaseF treated (27 samples, 27 RAW files, file name: *Number_PNGaseF_Replicate_Protein*)

Glycoform analyses
- Batch 1 glycoform analysis (27 samples, 54 wiff/scan files, file name: *Batch 1_Number_Replicate_Protein*)
- Batch 2 glycoform analysis (27 samples, 54 wiff/scan files, file name: *Batch 2_Number_Replicate_Protein*)
- Sialidase Treatment Validation (18 samples, 36 wiff/scan files, file name: *Sialidase_Number _Protein_ Sialidase or Control*)

SEARCH FILES

Proteome Discoverer glycan occupancy searches (27 files)

- Validated Peptide Spectrum Matches (27 text files, file name: *PD_Protein_Replicate_TargetPeptideSpectrumMatch_validated*)

Byonic glycoform searches (99 files)

Individual Byonic Excel Results files
- Byonic Results Batch 1 (27 excel files. file name: Byonic Batch 1_*Number _Replicate_Protein*)
- Byonic Results Batch 2 (27 excel files: file name: Byonic Batch 2_*Number _Replicate_Protein*)
- Byonic Results Occupancy Validation (27 excel files, file name: *Byonic_Occupancy Validation_Protein_Replicate*)
- Byonic Results Sialidase Treatment (18 excel files, file name: *Byonic_Sialidase_Number_Protein_Sialidase or Control*)

SEQUENCE FILES (2 files)

Glycan databases
- N-linked glycan database (1 text file; *N-linked glycan database*)
- O-linked glycan database (1 text file; *O-linked glycan database*)

QUANT FILES (9 files)

Skyline input library and output quantification files (6 files)

- Skyline Input (3 excel files, file name: *Skyline Input_Batch 1 or Batch 2 or Sialidase*)
- Skyline Output (3 csv files, file name: *Skyline Output_Batch 1 or Batch 2 or Sialidase*)

GlypNiro Output Files (3 files)

- GlypNiro Output (3 excel files, file name: *GlypNiro Output_Batch 1 or Batch 2 or Sialidase*)
